# Supplementary material for: Cryo-EM analyses of dimerized spliceosomes provide new insights into the functions of B complex proteins
Source: EMBO J. 2024 Feb 21;43(6):1065–88. doi: 10.1038/s44318-024-00052-1 (PMC10943123; doi:10.1038/s44318-024-00052-1)
Supplement: Supplementary file 1 — Appendix [file 44318_2024_52_MOESM1_ESM.pdf]

Table of Contents

Appendix Figure S1 ………………………………………………………… 2

Appendix Table S1 ………………………………………………………… 4

Appendix Table S2 ………………………………………………………… 7

Appendix Table S3 ………………………………………………………… 10

Appendix Table S4 ………………………………………………………… 11

Appendix Table S5 ………………………………………………………… 13

References …………………………………………………………….. 14

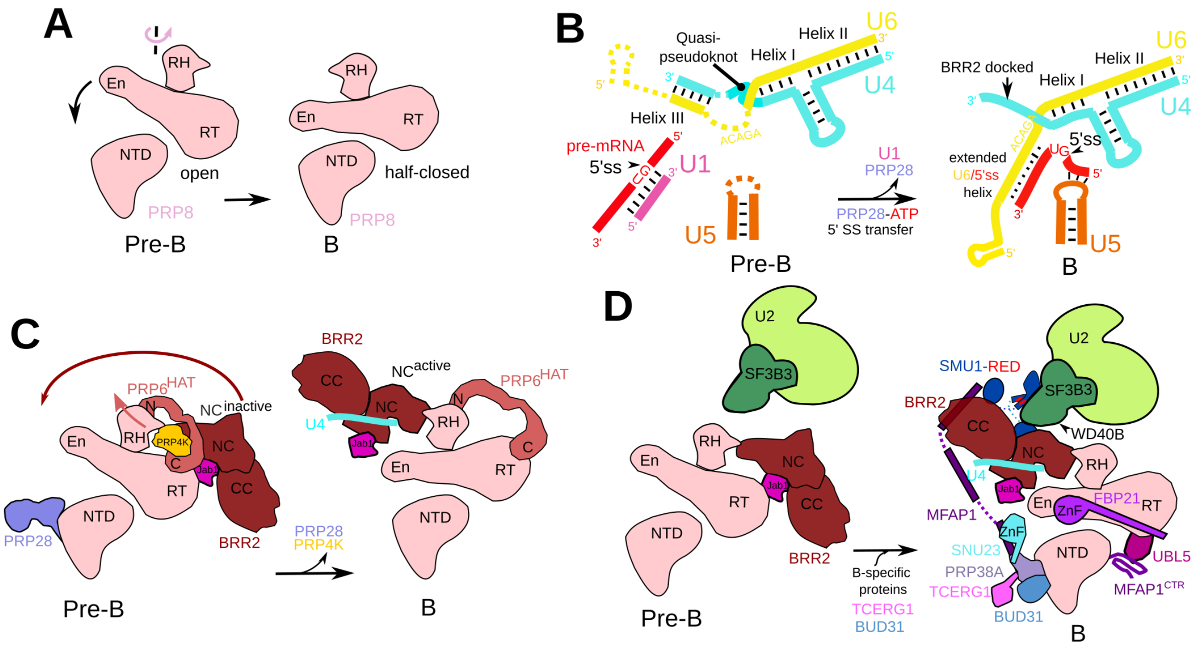

Appendix Figure S1. Structural changes during the spliceosomal pre-B to B complex transition. **A**. Cartoon of the conformational change in PRP8 during the transformation of the pre-B complex into B. For simplicity only selected PRP8 domains are shown. Movement of the PRP8 endonuclease-like (En) domain (indicated by an arrow) toward the PRP8 N-terminal domain (NTD) converts the PRP8 open conformation to a half-closed one in the B complex. During the pre-B to B transition, the PRP8 RNase H (RH) domain also rotates ca 180 degrees. RT, reverse transcriptase-like domain. **B**. Schematic showing the rearrangements in the U4/U6 RNA-RNA network and the transfer of the 5'ss from U1 to U6 during B complex formation. In the pre-B complex, the 5' end of the U1 snRNA base pairs with nucleotides at or adjacent to the 5'ss GU dinucleotide. During B complex formation, the DEAD-box helicase PRP28 disrupts the U1/5'ss interaction and facilitates the handover of the 5'ss to the U6 snRNA, leading to formation of an extended U6/5'ss helix. At the same time, nucleotides in loop 1 of the U5 snRNA base pair with pre-mRNA nucleotides directly upstream of the 5'ss GU at the 3' end of the 5' exon. See Fig. EV1H for a more comprehensive depiction of the RNA-RNA network in the human B complex. In the human pre-B complex, the U4 and U6 snRNAs, not only are extensively based-paired via helix I and II, but also form an additional helix (U4/U6 helix III) and the U4 snRNA forms a so-called quasi-pseudoknot. During B complex formation the latter and U4/U6 helix III are disrupted, and U6 nucleotides involved in helix III formation now form part of the extended U6/5'ss helix. **C**. Cartoon showing the large-scale translocation of the BRR2 helicase domain, comprised of an N-terminal helicase cassette (NC) and C-terminal helicase cassette (CC), during B complex formation. The BRR2 helicase domain is translocated from its position close to the PRP8 reverse transcriptase-like domain in pre-B to the PRP8 endonuclease-like domain in the B complex. In pre-B, the U4 snRNA binding site in the RecA1 and RecA2 domains of the BRR2 N-terminal cassette is blocked by the C-terminal tail of PRP8. In the B complex, the U4 snRNA has bound the RecA domains, leading to an active BRR2

conformation, but additional mechanisms involving other B complex proteins prevent BRR2 from unwinding the U4/U6 helices. The N-terminal HAT repeats of PRP6 are also repositioned during the transformation of pre-B to B, which potentially is triggered by PRP6 phosphorylation by PRP4 kinase (PRP4K). **D**. The B-specific proteins are recruited, together with TCERG1 and BUD13, during B-complex formation. See Appendix Table S1, for a description of the roles of the B-specific proteins in the B complex. The structural rearrangements and compositional changes described in the legend are based on this study and previous cryo-EM studies elucidating the structure of the human pre-B and B complexes (Bertram *et al*, 2017; Charenton *et al*, 2019; Zhan *et al*, 2018).

Appendix Table S1. Roles of the B-specific and other selected proteins in the human B complex. The roles of the listed proteins are based primarily on this study and previous cryo-EM studies of the human B complex (Bertram *et al*, 2017; Zhan *et al*, 2018).

SMU1	Bridges BRR2 and SF3B3, and helps to stabilize BRR2's new position after its translocation. It also bridges the two B complex protomers at their interface in the B dimer.
RED	Based on crosslinks, RED latches onto U2 proteins and PRP8, including the PRP8 N-terminal domain (NTD), likely stabilizing the U2 snRNP interaction with the tri-snRNP.
PRP38	Acts as a binding hub for the B-specific proteins MFAP1 and SNU23, and tethers them to the PRP8 NTD. PRP38 and associated proteins help to stabilize the half-closed conformation of PRP8.
SNU23	Stabilizes the extended part of the U6/5'ss helix by direct contacts or indirectly via interacting with the PRP8 RH-Jab1 linker. SNU23 interacts with the BRR2 N-terminal helicase cassette ($BRR2^{NC}$) and tethers it to the U6/5'ss helix.
FBP21	Binds to the U6/5'ss helix via its zinc finger and to the RecA2 domain of $BRR2^{NC}$ via its N-terminal α-helix, and thereby likely inhibits BRR2's movement towards U4/U6 helix I. It also binds via its C-terminal region to BRR2's C-terminal helicase cassette, thereby inhibiting BRR2's helicase activity.
MFAP1 & UBL5	MFAP1 bridges via a long $\alpha\text{-helix}^{291\text{-}313}$ to PRP38, and SNU23, via $\alpha\text{-helix}^{215\text{-}255}$ to $BRR2^{NC}$ and via $\alpha\text{-helix}^{142\text{-}166}$ to the WD40 domain of SMU1B, thereby likely stabilizing BRR2 in its new position after translocation. Based on crosslinks, the N-terminal region latches on U2 SF3B1, thus MFAP1 connects SMU1, BRR2 and the 5' domain of U2 with the RNP core of the B complex. A large part of MFAP1's C-terminal region (aa 215-393) forms a globular domain that binds UBL5, and together with it, forms a 5' exon- binding channel. Both proteins also stabilize the 5' exon/U5 loop 1 base pairing interaction.
DIM1	The U5 protein DIM1 recognizes the 5'ss nucleotide U+2. It also contacts, together with FBP21, U6 nts 47-48. As these U6 nts stabilize the quasi-pseudoknot structure of U4 nts 63-68 in the U4/U6.U5 tri-snRNP, DIM1 may thus help to dissolve the quasi-pseudoknot structure of U4 during the pre-B to B transition.
TCERG1	The six C-terminal FF domains of TCERG1 act as a brace contacting the PRP8 reverse transcriptase-like (RT) domain, SNU114 and, in conjunction with BUD31, the 5' end of U6 snRNA, likely stabilizing this part of the B complex during the BRR2-mediated remodeling of the spliceosome.

PRP8	Acts as a large scaffold that binds numerous B complex proteins and RNA regions. The PRP8 NTD clamps the major stems of the U5 snRNA and stably interacts with SNU114. It also provides docking sites for the extended U6/5'ss helix, and for the PRP38/SNU23/MFAP1 protein complex, as well as BUD13 and the TCERG1 FF1 domain. The PRP8 En domain interacts with the PRP8 Jab1 domain, which in turn stably docks to the N-terminal helicase cassette of BRR2. The PRP8 RH domain interacts with BRR2, SNU66 and the N-terminal HAT domain of PRP6. Amino acid side chains of two loops of the PRP8 Linker region recognize the 5'ss nucleotides G+1 and G-1. The PRP8 helical bundle binds DIM1. Several regions of the PRP8 Large domain interact with U4/U6 RNP proteins.
BRR2	A DEXH-box RNA helicase that unwinds the U4/U6 helix during B^{act} formation. The RecA domains of the N-terminal helicase cassette bind a single stranded region of the U4 snRNA directly upstream of the U4/U6 helix 1.
PRP6	The PRP6 HAT domain bridges the U4/U6 and U5 snRNPs; that is, the C-terminal HAT repeats bind to the U4/U6 proteins PRP4, SNU13, PRP3 and PRP31, while the N-terminal-most TPR repeats interact with the PRP8 RH and RT domains. The "elbow region" within the N-terminal HAT domain, together with the SNU66 α-helix$^{200\text{-}219}$ forms part of the central dimerization interface of the two protomers in the B dimer.
PRP4	The PRP4 WD40 domain bridges the U4/U6 proteins SNU13 and PRP6, as well as the C-terminal ferredoxin-like domain of PRP3. The N-terminal helical bundle of PRP4 binds PPIH, BRR2, and the central region of SF3A1.
SNU66	Several N-terminally-located α-helices of SNU66 connect the N-terminal HAT domain of PRP6 with the PRP8 RT, RH and NTD domains, and likely stabilize these domains in the B complex after their rearrangements during the pre-B to B transition.
PPIH	PPIH binds to the N-terminal region of PRP4 and provides a major docking site for the long N-terminal α-helix of FBP21. It also contacts PRP3.
SF3B1	The SF3B1 HEAT domain clamps the extended U2/BS helix and acts as a binding platform for several other SF3b complex proteins.
SF3B6	The SF3B6 RRM is bound at the C-terminal region of the SF3B1 HEAT domain and is located near U2/U6 helix II.
SF3A1	SF3A1 connects the U2 snRNP with the tri-snRNP core via its central tri-snRNP-interacting region comprised of amino acids 409-489. This region contains three α-helices that dock to PRP4's N-terminal helical bundle and PPIH, bind to the 5' stem-loop of U4 snRNA, and interact via its most C-terminally located α-helix with the U5 protein DIM1.

SF3A2	The SF3A2 β-sandwich domain and the SF3B4 RRM1 domain chaperon the intron region directly upstream of nucleotides forming the branch site helix. Based on crosslinks, the β-sandwich domain may also function as a docking site for the RRM1 of the hnRNP A1 protein.

Appendix Table S2. **Protein composition of hB complexes.** Proteins were identified by nano UHPLC-ESI MS with at least 2 unique peptides (peptide FDR 0.05%). PSM stands for peptide-spectrum match. The 90 most abundant proteins are shown as judged from a ratio of PSMs to protein molecular weight. PSMs are the sum of three technical replicates. Asterisk marks the recombinant protein used for affinity purification of hB.

Name	Uniprot AC	kDa	Gene name	PSMs
**Sm proteins**				
SMB/B'	P14678	24.6	SNRPB	1856
SMD1	P62314	13.3	SNRPD1	880
SMD2	P62316	13.5	SNRPD2	1038
SMD3	P62318	13.9	SNRPD3	1419
SME	P62304	10.8	SNRPE	1293
SMF	P62306	9.7	SNRPF	504
SMG	P62308	8.5	SNRPG	593
**U1 snRNP**				
U1-70K	P08621	51.6	SNRNP70	1590
U1-A	P09012	31.3	SNRPA	1018
U1-C	P09234	17.4	SNRPC	381
**17S U2 snRNP**				
U2A'	P09661	28.4	SNRPA1	1466
U2B"	P08579	25.5	SNRPB2	1407
SF3A1	Q15459	88.9	SF3A1	3344
SF3A2	Q15428	49.3	SF3A2	1285
SF3A3	Q12874	58.9	SF3A3	3137
SF3B1	O75533	145.8	SF3B1	7278
SF3B2	Q13435	100.2	SF3B2	5545
SF3B3	Q15393	135.6	SF3B3	6896
SF3B4	Q15427	44.4	SF3B4	1321
SF3B5	Q9BWJ5	10.1	SF3B5	1130
SF3B6	Q9Y3B4	14.6	SF3B6	465
PHF5A	Q7RTV0	12.4	PHF5A	390
**17S U2 related**				
DDX42	Q86XP3	103.0	DDX42	4409
DHX15	O43143	90.9	DHX15	3912
PUF60	Q9UHX1	59.9	PUF60	1765
SPF30	O75940	26.7	SMNDC1	592
U2AF35	Q01081	27.9	U2AF1	1838
U2AF65	P26368	53.5	U2AF2	3683
**U5 snRNP**				
PRP8	Q6P2Q9	273.6	PRPF8	16978
BRR2	O75643	244.5	SNRNP200	17266
SNU114	Q15029	109.4	EFTUD2	9283

Name	Uniprot AC	kDa	Gene name	PSMs
U5-40K	Q96DI7	39.3	SNRNP40	3589
PRP6 (102K)	O94906	106.9	PRPF6	3471
DIM1 (15K)	P83876	16.8	TXNL4A	892
**LSm proteins**				
LSM2	Q9Y333	10.8	LSM2	727
LSM3	P62310	11.8	LSM3	622
LSM4	Q9Y4Z0	15.4	LSM4	610
LSM5	Q9Y4Y9	9.9	LSM5	207
LSM6	P62312	9.1	LSM6	261
LSM7	Q9UK45	11.6	LSM7	478
LSM8	O95777	10.4	LSM8	725
**U4/U6 snRNP**				
PRP3	O43395	77.6	PRPF3	3158
PRP4	O43172	58.5	PRPF4	3200
PPIH (20K)	O43447	19.2	PPIH	912
PRP31	Q8WWY3	55.5	PRPF31	2088
NHP2L1 (15.5K)	P55769	14.2	SNU13	358
**U4/U6.U5 tri-snRNP**				
SNU66	O43290	90.3	SART1	3688
SAD1	Q53GS9	65.4	USP39	1590
**B-specific proteins**				
FBP21	O75554	42.5	WBP4	944
MFAP1	P55081	52.0	MFAP1	1181
NPW38BP	Q9Y2W2	70.0	WBP11	1974
PQBP1	O60828	30.5	PQBP1	1059
PRP38A	Q8NAV1	37.5	PRPF38A	1543
RED	Q13123	65.6	IK	2572
SMU1 (fSAP57)	Q2TAY7	57.5	SMU1	4133
SNU23 (ZMAT2)	Q96NC0	23.6	ZMAT2	968
UBL5	Q9BZL1	8.5	UBL5	387
**Cap binding complex**				
NCBP1	Q09161	91.8	NCBP1	6135
NCBP2	P52298	18.0	NCBP2	557
**SR proteins**				
SRSF1	Q07955	27.8	SRSF1	2242
SRSF2	Q01130	25.5	SFRS2	736
SRSF3	P84103	19.3	SFRS3	566
SRSF7	Q16629	27.4	SRSF7	670
**hnRNP**				
HNRNPA1	P09651	38.7	HNRNPA1	2365
HNRNPA2/B1	P22626	37.4	HNRPA2B1	1389
HNRNPA3	P51991	39.6	HNRNPA3	834
**hPRP19/CDC5L complex**				

Name	Uniprot AC	kDa	Gene name	PSMs
PRP19	Q9UMS4	55.2	PRPF19	4711
CDC5L	Q99459	92.2	CDC5L	2869
SPF27	O75934	26.0	BCAS2	1001
PLRG1	O43660	57.2	PLRG1	2116
CTNNBL1	Q8WYA6	65.1	CTNNBL1	2029
**hPRP19/CDC5L related**				
BUD31	P41223	17.0	BUD31	774
SKIP	Q13573	61.5	SNW1	1471
SYF2	O95926	28.7	SYF2	581
**Intron binding complex (IBC)**				
AQR	O60306	171.3	AQR	4283
ISY1	Q9ULR0	33.0	KIAA1160	773
SYF1	Q9HCS7	100.0	XAB2	2340
**Other spliceosomal proteins**				
TCERG1 (CA150)	O14776	123.9	TCERG1	2960
BUB3	O43684	37.2	BUB3	1056
CCAR1 (FLJ10839)	Q8IX12	132.8	CCAR1	3004
CCDC12	Q8WUD4	19.2	CCDC12	455
ERH	P84090	12.3	ERH	254
FUS	P35637	53.4	FUS	1163
PPWD1 (KIAA0073)	Q96BP3	73.6	PPWD1	1535
PRP38B	Q5VTL8	64.5	PRPF38B	1437
RBM39 (RNPC2, CAPER)	Q14498	58.5	RBM39	2236
SMN	P63162	24.6	SNRPN	1838
SRRT (ASR2B)	Q9BXP5	100.0	SRRT	5284
TRIR (fSAP18)	Q9BQ61	18.4	TRIR	414
MBP-MS2 (recombinant)*		56.9		11600

Appendix Table S3 Cryo-EM data collection, refinement and validation statistics for hB complexes.

	Human B complex			
	B complex dimer (EMD-19063)	B complex protomer (EMD-18529) (PDB:8QO9)	tri-snRNP region (EMD-18225) (PDB: 8Q7N)	BRR2 region (EMD-19062)
Data collection and processing				
Magnification	120,700	120,700	120,700	120,700
Voltage (kV)	300	300	300	300
Electron exposure (e⁻/Å^2)	48	48	48	48
Defocus range (μm)	1-3	1-3	1-3	1-3
Pixel size (Å)	1.16	1.16	1.16	1.16
Symmetry imposed	C1	C1	C1	C1
Initial particle images (no.)	~1 million	~1 million	~1 million	~1 million
Final particle images (no.)	25,833	50,321	251,564	251,564
Map resolution (Å)	15.0	5.3	3.1	4.2
FSC threshold	0.143	0.143	0.143	0.143
Map resolution range (Å)				
Tri-snRNP region	15-20	4.5-6	2.9-3.5	---
U2 region	20-30	10-15	10-15	---
BRR2 region	20-30	10-15	8-15	4-8
Refinement				
Initial model used (PDB code)	n/a	6AHD	6AHD	n/a
Model resolution (Å)	---	---	3.3	---
FSC threshold	---		0.5	---
Model resolution range (Å)	---	---	3.3	---
Map sharpening *B* factor (Å^2)	---	-200	-104	-215
Model composition				
Non-hydrogen atoms	---	87,345	56,046	---
Protein residues	---	15,647	6,666	---
Ligands	---	0	0	---
B factors (Å^2)				
Protein	---	---	76.03	---
Ligand	---	---		
R.m.s. deviations				
Bond lengths (Å)	---	---	0.0178	---
Bond angles (°)	---	---	1.44	---
Validation				
MolProbity score	---	---	1.88	---
Clashscore	---	---	7.43	---
Poor rotamers (%)	---	---	0.85	---
Ramachandran plot				
Favored (%)	---	---	92.41	---
Allowed (%)	---	---	7.42	---
Disallowed (%)	---	---	0.17	---

Appendix Table S4. Summary of modeled proteins and RNAs in hB complexes.

Sub-complexes	Protein/RNA	Chain ID	UniProt ID	Modeled Region/domain	Template	modeling approach
U2 snRNP	U2 snRNA	2	N.A.	3-14; 33-65; 97-107; 147-184	6AHD	docked
				29-32	7Q4O	docked
	U2 A’	2A	P09661	2-163	6FF7	docked
	U2 B’’	2B	P08579	3-84	6FF7	docked
	SF3B1	B1	O75533	457-1304	6FF7	docked
				394-415	7Q4O	docked
	SF3B2	B2	Q13435	458-602 ; 604-667	6FF7	docked
	SF3B3	B3	Q15393	1-645 ; 663-691 ; 695-830 ; 834-1068 ; 1078-1217	6FF7	docked
	SF3B4	B4	Q15427	12-89	6FF7	docked
	SF3B5	B5	Q9BWJ5	12-80	6FF7	docked
	SF3B6	B6	Q9Y3B4	12-101	7Q4O	docked
	PHF5A	BP	Q7RTV0	2-101	6FF7	docked
	SmB,D1,D2, D3,E,F,G	2b, 21, 22, 23,2e, 2f, 2g	P14678, P62314, P62316, P62318, P62304, P62306, P62308	Sm fold	6FF7	docked
	SF3A1	7	Q15459	160-282	6FF7	docked
				422-447; 455-473	6QX9	docked and adjusted
				409-421; 448-454; 474-489	N.A.	de novo modelling
	SF3A2	8	Q15428	41-85 ; 104-126 ; 133-209	6FF7	docked
	SF3A3	9	Q12874	1-229 ; 279-362 ; 393-462	6FF7	docked
U4/U6 di-snRNP	U4 snRNA	4	N.A.	1-62; 68-96; 105-133; 138-145	6AHD	docked and adjusted
	SmB,D1,D2, D3,E,F,G	4b, 41, 42, 43, 4e, 4f, 4g	P14678, P62314, P62316, P62318, P62304, P62306, P62308	Sm fold	6AHD	docked
	U6 snRNA	6	N.A.	1-78	6AHD	docked and adjusted
				85-96; 102-105	6AHD	docked
	LSm2, LSm3, LSm4, LSm5, LSm6, LSm7, LSm8	62, 63, 64, 65, 66, 67, 68	Q9Y333, P62310, Q9Y4Z0, Q9Y4Y9, P62312, Q9UK45, O95777	Sm fold	6AHD	docked
	PRP3	J	O43395	385-395;412-427	AlphaFold2	docked
				413-606; 627-683	6AHD	docked and adjusted
	PRP4	F	O43172	81-152; 164-522	AlphaFold2	docked and adjusted
	PRP31	L	Q8WWY3	52-80; 86-347; 351-432	6AHD	docked and adjusted
	PPIH	W	O43447	9-177	6AHD	docked and adjusted
	SNU13	M	P55769	5-128	6AHD	docked and adjusted
Tri-snRNP	SNU66	S	O43290	148-191; 198-219; 359-375	AlphaFold2	docked and adjusted
				252-358	AlphaFold2	docked and adjusted
	U5 snRNA	5	N.A.	3-117	6AHD	docked and adjusted

U5 snRNP	PRP8	A	Q6P2Q9	58-664; 679-2026; 2068-2316	6AHD	docked and adjusted
				2038-2067	AlphaFold2	docked and adjusted
	BRR2	B	O75643	433-2125	6AHD	docked and adjusted
	SNU114	C	Q15029	105-956	6AHD	docked and adjusted
	DIM1	D	P83876	2-142	6AHD	docked and adjusted
	U5-40K	E	Q96DI7	58-356	6AHD	docked
	SmB,D1,D2, D3,E,F,G	5b, 51, 52, 53, 5e, 5f, 5g	P14678, P62314, P62316, P62318, P62304, P62306, P62308	Sm fold	6AHD	docked
	PRP6	N	O94906	8-37; 136-140	N.A.	de novo modelling
				141-208; 247-257; 265-941	6AHD	docked and adjusted
B-specific	SMU1	v,w	Q2TAY7	3-188	6Q8I	docked
				204-513	6AHD	docked
	RED	x,y	Q13123	207-211; 230-245; 250-257	6Q8I	docked
	SNU23	r	Q96NC0	16-41	AlphaFold2	docked
				45-133	AlphaFold2	docked and adjusted
	UBL5	s	Q9BZL1	1-73	AlphaFold2	docked and adjusted
	MFAP1	K	P55081	141-174; 214-256;	AlphaFold2	docked
				271-314	AlphaFold2	docked and adjusted
				315-405	AlphaFold2	docked and adjusted
	PRP38	I	Q8NAV1	1-184	AlphaFold2	docked and adjusted
	FBP21	X	O75554	1-8	N.A.	de novo modelling
				9-82	6AHD	docked and adjusted
Other	TCERG1	T	O14776	657-845; 856-1080	AlphaFold2	docked and adjusted
	SRSF1	z	Q07955	122-195	7ABG	docked
	BUD31	Q	P41223	3-144	6FF4	docked and adjusted
	Pre-mRNA	Z	N.A.	49-79 ('-10' - '+21')	N.A.	de novo modelling
				144-158	6AHD	docked

Appendix Table S5. Summary of newly-modeled or more accurately docked/modeled protein and RNA regions in our hB complex. Improvements are based on comparisons with previously published hB complex cryo-EM structures (Bertram *et al*, 2017; Zhan *et al*, 2018). "Docking" refers to the placement of a known protein structure (as a rigid body) into the low-resolution EM density map. "Located for the first time" indicates that a known protein structure was not included in previous hB complex models but could be fit (i.e., docked) for the first time into our low-resolution EM density map. "Modeled for the first time" means that the atomic model was built into high-resolution EM density based on existing crystal structures or AlphaFold predictions.

	Protein/RNA	Modeled Region/domain	
U2 snRNP	U2 snRNA	3-14; 29-65	Improved resolution allows more accurate docking
	SF3B1	457-1304<br>394-415	
	SF3B2	458-602; 604-667	
	SF3B3	1-645; 663-691; 695-830 ; 834-1068; 1078-1217	
	SF3B4	12-89	
	SF3B5	12-80	
	PHF5A	2-101	
	SF3B6	12-101	Located for the first time in the human B complex
	SmB,D1,D2,D3,E,F,G	Sm fold	Improved resolution allows more accurate docking
	SF3A1	160-282	
		422-447; 455-473 409-421; 448-454; 474-489	Modeled for the first time in the human B complex
	SF3A2	41-85 ; 104-126 ; 133-209	Improved resolution allows more accurate docking
	SF3A3	1-229 ; 279-362 ; 393-462	
	U2 snRNA	97-107; 147-184	
	U2 A'	2-163	
	U2 B''	3-84	
U4/U6 di-snRNP	U4 snRNA	1-62; 68-76	Improved resolution allows more accurate modeling
		77-96 ;105-133; 138-145	--
	SmB,D1,D2,D3,E,F,G	Sm fold	
	U6 snRNA	1-43; 50-78	Improved resolution allows more accurate modeling
		44-49	Previously incorrectly-modeled region was corrected
		85-96	Improved resolution allows more accurate docking
		102-105	--
	LSm2, LSm3, LSm4, LSm5, LSm6, LSm7, LSm8	Sm fold	
	PRP3	385-395;412-427	Improved resolution allows more accurate docking
		413-606; 627-683	Improved resolution allows more accurate modeling
	PRP4	81-152; 164-522	
	PRP31	52-80; 86-347; 351-432	
	PPIH	9-177	Improved resolution allows more accurate docking
	SNU13	5-128	Improved resolution allows more accurate modeling
Tri-snRNP	SNU66	148-191; 198-219; 252-358; 359-375	
U5 snRNP	U5 snRNA	3-117	
	PRP8	58-664; 679-2026; 2068-2316	
		2038-2067	Modeled for the first time in the human B complex
	BRR2	433-1288	Improved resolution allows more accurate modeling
		1289-2125	Improved resolution allows more accurate docking
	SNU114	105-956	Improved resolution allows more accurate modeling
	DIM1	2-142	
	U5-40K	58-356	Improved resolution allows more accurate docking
	SmB,D1,D2,D3,E,F,G	Sm fold	
	PRP6	8-37; 136-140	Modeled for the first time in the human B complex
		141-208; 247-257	Improved resolution allows more accurate modeling
		265-941	Improved resolution allows more accurate docking
B-specific	SMU1	3-188<br>204-513	Located for the first time in the human B complex
	RED	207-211; 230-245; 250-257	
	SNU23	16-41	
		45-80	Modeled for the first time in the human B complex
		81-133	Improved resolution allows more accurate modeling
	UBL5	1-73	
	MFAP1	141-174	
		214-256; 271-314	Located for the first time in the human B complex
		315-405	Modeled for the first time in the human B complex
	PRP38	1-184	Improved resolution allows more accurate modeling
	FBP21	1-7	Modeled for the first time in the human B complex
		8-82	Improved resolution allows more accurate modeling
Other	TCERG1	657-845; 856-1080	Located for the first time in the human B complex
	SRSF1	122-195	
	BUD31	3-144	Modeled for the first time in the human B complex
	Pre-mRNA	49-53 (-10 to -6 relative to 5'ss)	Previously incorrectly-modeled region was corrected
		54-79 (-5 to +21 relative to 5'ss)	Improved resolution allows more accurate modeling
		144-158	Improved resolution allows more accurate docking

References

Bertram K, Agafonov DE, Dybkov O, Haselbach D, Leelaram MN, Will CL, Urlaub H, Kastner B, Lührmann R, Stark H (2017) Cryo-EM structure of a pre-catalytic human spliceosome primed for activation. *Cell* 170: 701-713

Charenton C, Wilkinson ME, Nagai K (2019) Mechanism of 5' splice site transfer for human spliceosome activation. *Science* 364: 362-367

Zhan X, Yan C, Zhang X, Lei J, Shi Y (2018) Structures of the human pre-catalytic spliceosome and its precursor spliceosome. *Cell Res* 28: 1129-1140
