# Supplementary material for: Cryo-EM analyses of dimerized spliceosomes provide new insights into the functions of B complex proteins
Source: EMBO J. 2024 Feb 21;43(6):1065–88. doi: 10.1038/s44318-024-00052-1 (PMC10943123; doi:10.1038/s44318-024-00052-1)
Supplement: Supplementary file 4 — Expanded View Figures [file 44318_2024_52_MOESM4_ESM.pdf]

Expanded View Figures

Figure EV1. Cryo-EM and image-processing of the hB complex.

(**A**) RNA composition of purified B complex dimers. Human (h) B complexes were affinity-purified and RNA from the peak gradient fractions was isolated, separated on a NuPAGE gel, and visualized by staining with SyBr gold. U1 snRNA, that is no longer base paired to the 5′ss of the MINX pre-mRNA is still present, together with U2, U4, U5 and U6 snRNA, and the MINX pre-mRNA. It is likely that, under our low salt purification conditions, U1 snRNP remains bound via protein–protein interactions (i.e., in the poorly resolved, globular EM densities). (**B**) Cryo-EM computation sorting scheme for the hB complexes. All major image-processing steps are depicted. (**C**) Representative cryo-EM 2D class averages of the human B complex dimers. (**D**) Local resolution estimation of the tri-snRNP core region of the B complex. (**E**) Orientation distribution plot for the particles contributing to the reconstruction of the tri-snRNP core region. (**F**) Fourier shell correlation (FSC) values for the listed parts of the B complex, indicate a resolution of 3.1 Å for the tri-snRNP core and 4.2 Å for the BRR2 region. (**G**) Map versus model FSC curves generated for the tri-snRNP core and BRR2 regions of hB using PHENIX mtriage. (**H**) Schematic of the RNA-RNA network in the hB complex. Red nucleotides are from the MINX pre-mRNA.

© The Author(s)

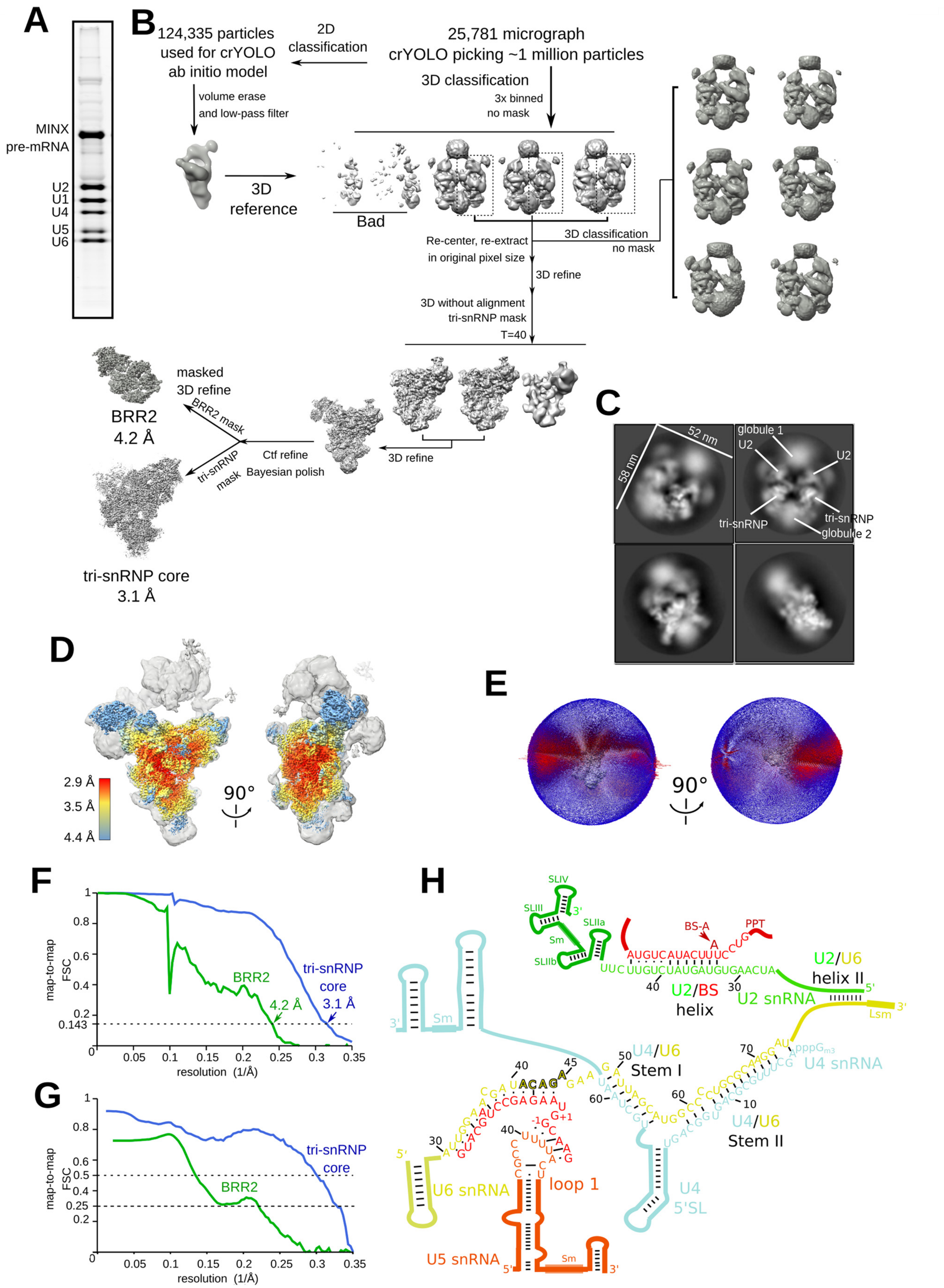
A
MINX pre-mRNA
U2
U1
U4
U5
U6
B
124,335 particles used for crYOLO ab initio model
2D classification
25,781 micrograph
crYOLO picking ~1 million particles
3D classification
3x binned no mask
volume erase and low-pass filter
3D reference
Bad
Re-center, re-extract in original pixel size
3D classification no mask
3D refine
3D without alignment tri-snRNP mask T=40
masked 3D refine
BRR2 mask
BRR2 4.2 Å
tri-snRNP mask
Ctf refine Bayesian polish
3D refine
tri-snRNP core 3.1 Å
C
52 nm
58 nm
globule 1
U2
U2
tri-snRNP
tri-snRNP
globule 2
D
2.9 Å
3.5 Å
4.4 Å
90°
E
90°
F
map-to-map FSC
BRR2
tri-snRNP core
4.2 Å
3.1 Å
0.143
resolution (1/Å)
G
map-to-map FSC
tri-snRNP core
BRR2
resolution (1/Å)
H
SLIV
SLIII
SLIIa
Sm
SLIIb
BS-A
PPT
AUGUCAUACUUUC
UUC UUGUCUAUGAUGUGAACUA
U2/U6 helix II
U2/BS helix
U2 snRNA
Lsm
U4/U6 Stem I
ACAG
pppGm3
U4 snRNA
U4/U6 Stem II
-1G
G+1
loop 1
U6 snRNA
U4 5'SL
U5 snRNA
Sm

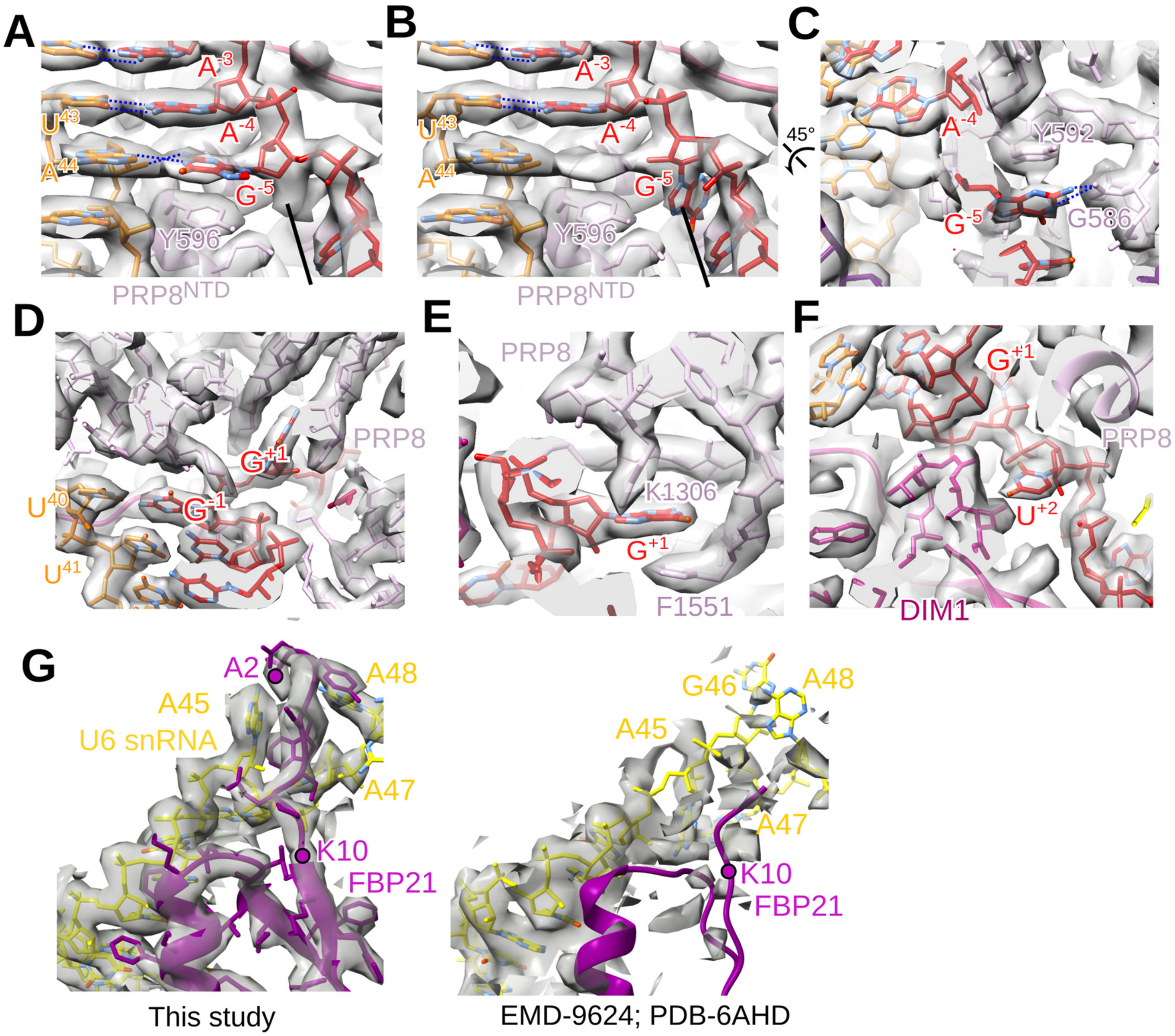

Figure EV2. Molecular interactions with the 5′ss nucleotides in hB.

(**A–C**) Two different conformations (panels **A** and **B**) of the base of G^{-5} of the 5′ exon and their stabilization by neighboring residues of the PRP8 N-terminal domain ($PRP8^{NTD}$). The fit of U5 snRNA (orange) and 5′ exon nucleotides (red), where numbers indicate the nucleotide position relative to the 5′ss GU dinucleotide at the 5′end of the intron, to the EM density is shown. Dashed lines, hydrogen bonds. Panel (**C**) shows a different view of the conformation shown in panel (**B**). In one conformation, G^{-5} base pairs with U5-A44 via two hydrogen bonds, one with the base of U5-A44, and the other with the preceding phosphate group. Moreover, the position of the G^{-5} base is further stabilized by stacking interactions with Y592 of $PRP8^{NTD}$ (panel **A**). In the alternate conformation, the G^{-5} base is flipped back by about 0.6 nm (panel **B**) and is stabilized by stacking interactions with Y592 and by hydrogen bonds with the protein main chain at G586 of $PRP8^{NTD}$ (panel **C**). The functional relevance of these alternative conformations, is not clear. However, after step 1 (i.e., in the human spliceosomal C complex), the base-paired conformation appears to be favored (Bertram et al, 2020), which would help to tether the cleaved 5′ exon to the spliceosome prior to exon ligation. (**D–F**) Fit of 5′ss nucleotides and neighboring RNAs and proteins to the hB EM density. (**G**) Fit of the N-terminus of FBP21, together with the U6 nts with which it interacts, into the hB EM density (left panel). Comparison with a previous hB complex model (PDB-GAHD) (Zhan et al, 2018), in which U6 snRNA nts 46–47 were incorrectly placed in the density occupied by the N-terminus of FBP21 (right panel). In the B complex from the yeast *S. cerevisiae*, the 5′ss and U6 ACAGA box region, as well as the 5′ exon and 5′ stem-loop of the U6 snRNA, appear to be organized somewhat differently compared to the hB complex (Plaschka et al, 2017; Bai et al, 2018).

© The Author(s)

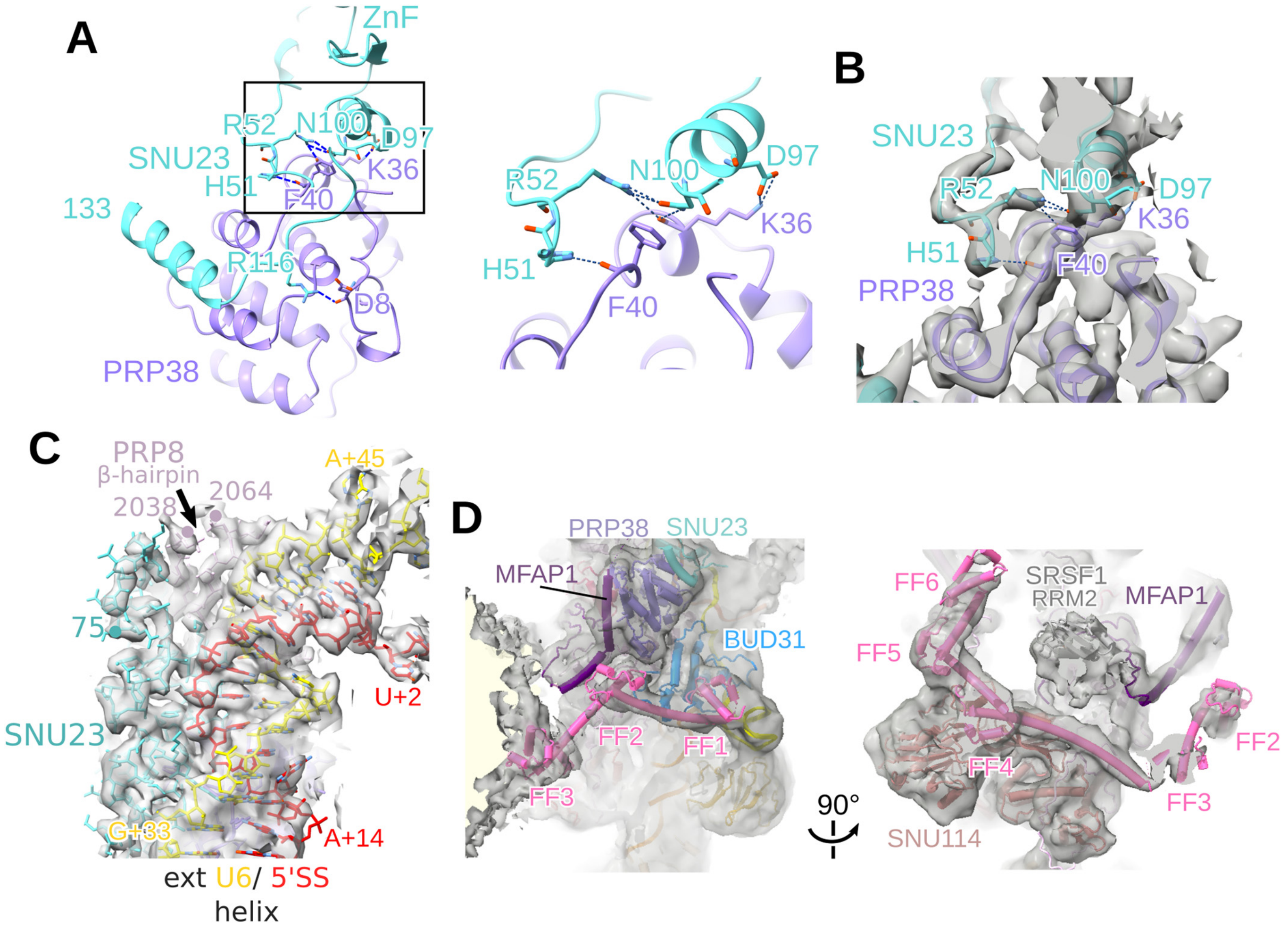

Figure EV3. Interface of the SNU23 zinc finger and PRP38 N-terminal region, and localization of TCERG1 and SRSF1.

(**A**) Interface between the SNU23 zinc finger (ZnF) and PRP38 N-terminal region. The boxed region is expanded in the right panel. (**B**) Fit of the SNU23 and PRP38 residues shown at the right in panel (**A**) into the hB EM density. (**C**) Fit of SNU23, the PRP8 RNase H-Jab1 linker and the U6/5′ss helix to the hB EM density. (**D**) Fit of the TCERG1 FF1-FF6 domains and SRSF1 RRM2 into the EM density of the hB monomer. Although the α-helix bridging FF2 and FF3 cannot be visualized in hB, fitting of the FF1-FF3 structure from pre-B^{act-1} into our B complex indicates that FF3 is located in a small globular density that directly contacts the lower globule 2.

 © The Author(s)

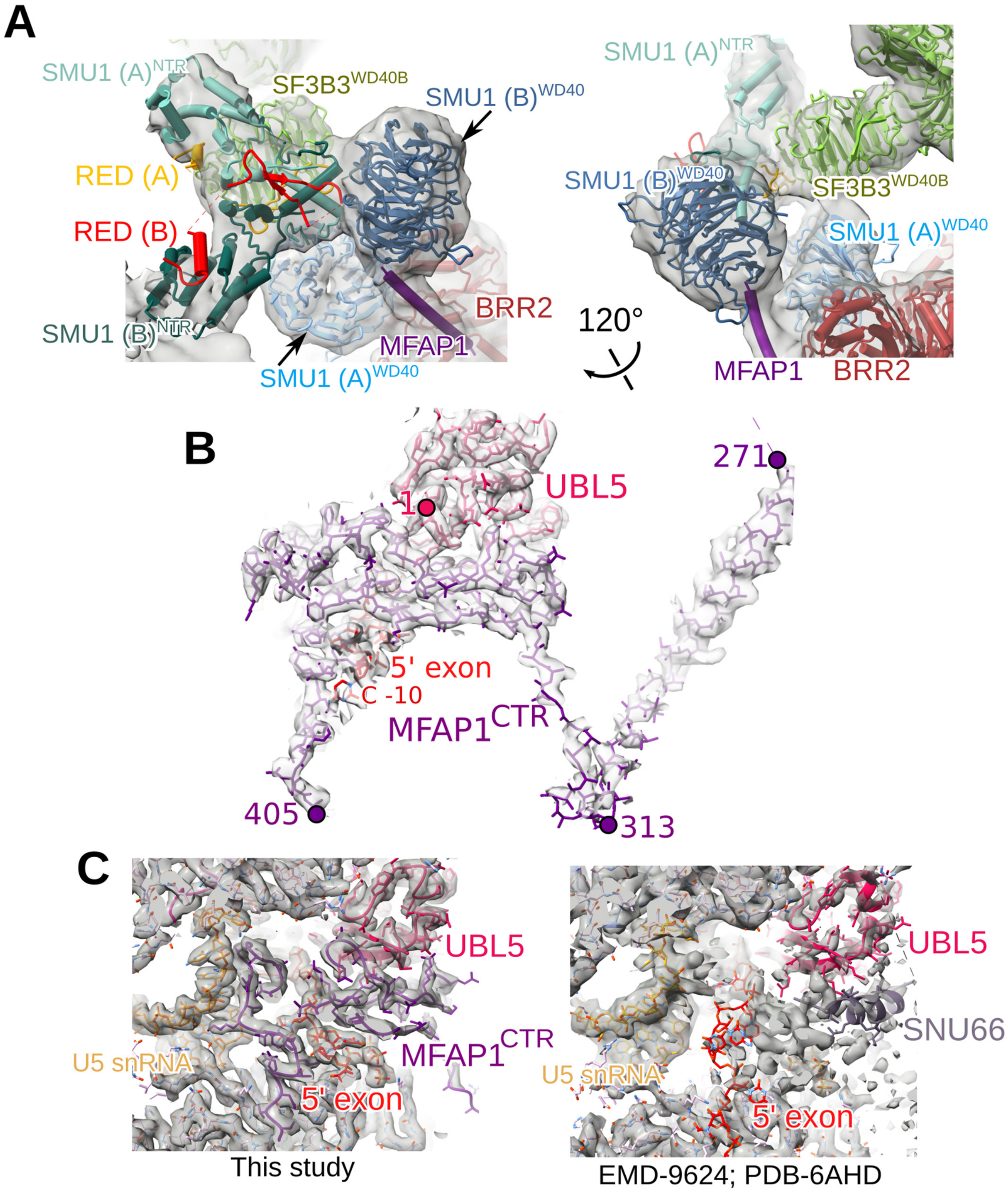
A
SMU1 (A)NTR
SF3B3WD40B
SMU1 (B)WD40
RED (A)
RED (B)
SMU1 (B)NTR
SMU1 (A)WD40
MFAP1
BRR2
120°
SF3B3WD40B
MFAP1
BRR2
B
271
UBL5
1
5' exon
C -10
MFAP1CTR
405
313
C
UBL5
MFAP1CTR
U5 snRNA
5' exon
This study
SNU66
EMD-9624; PDB-6AHD

Figure EV4. A heterotetrameric SMU1-RED complex is present in the human B complex.

(**A**) Fit of the SMU1 N-terminal region (NTR) and the WD40 domain of each SMU1 subunit (denoted **A** or **B**) of the SMU1 dimer, plus 2 copies of RED, into the hB EM density. The orientation of the individual NTR domains (**A** or **B**) cannot be determined unambiguously. That the B-specific proteins SMU1 and RED interact with each other was initially shown by co-precipitation experiments (Chung et al, 2009) and luciferase complementation assays (Fournier et al, 2014). The crystal structure of a minimal SMU1-RED complex from C. elegans and human, comprised of the SMU1 N-terminal region and a middle region of RED, showed that SMU1 dimerizes via its LiSH motif and a C-terminal α-helix, and that the dimerization module binds two copies of RED (Ashraf et al, 2019; Ulrich et al, 2016a). The human structure suggests that the SMU1 N-terminal region must first form a dimer before it can interact with two copies of the middle domain of RED (Ashraf et al, 2019). (**B**) Fit of MFAP1 helix 217–313 and its C-terminal region (CTR), as well as UBL5 and the 3′ end of the 5′ exon, to the hB EM density. (**C**) Fit of amino acids in the MFAP1 C-terminal region to the hB EM density. Comparison with a previous hB complex model (PDB-6AHD) (Zhan et al, 2018), in which an α-helix of SNU66 was incorrectly placed in the density occupied by the MFAP1 C-terminal region.

 © The Author(s)

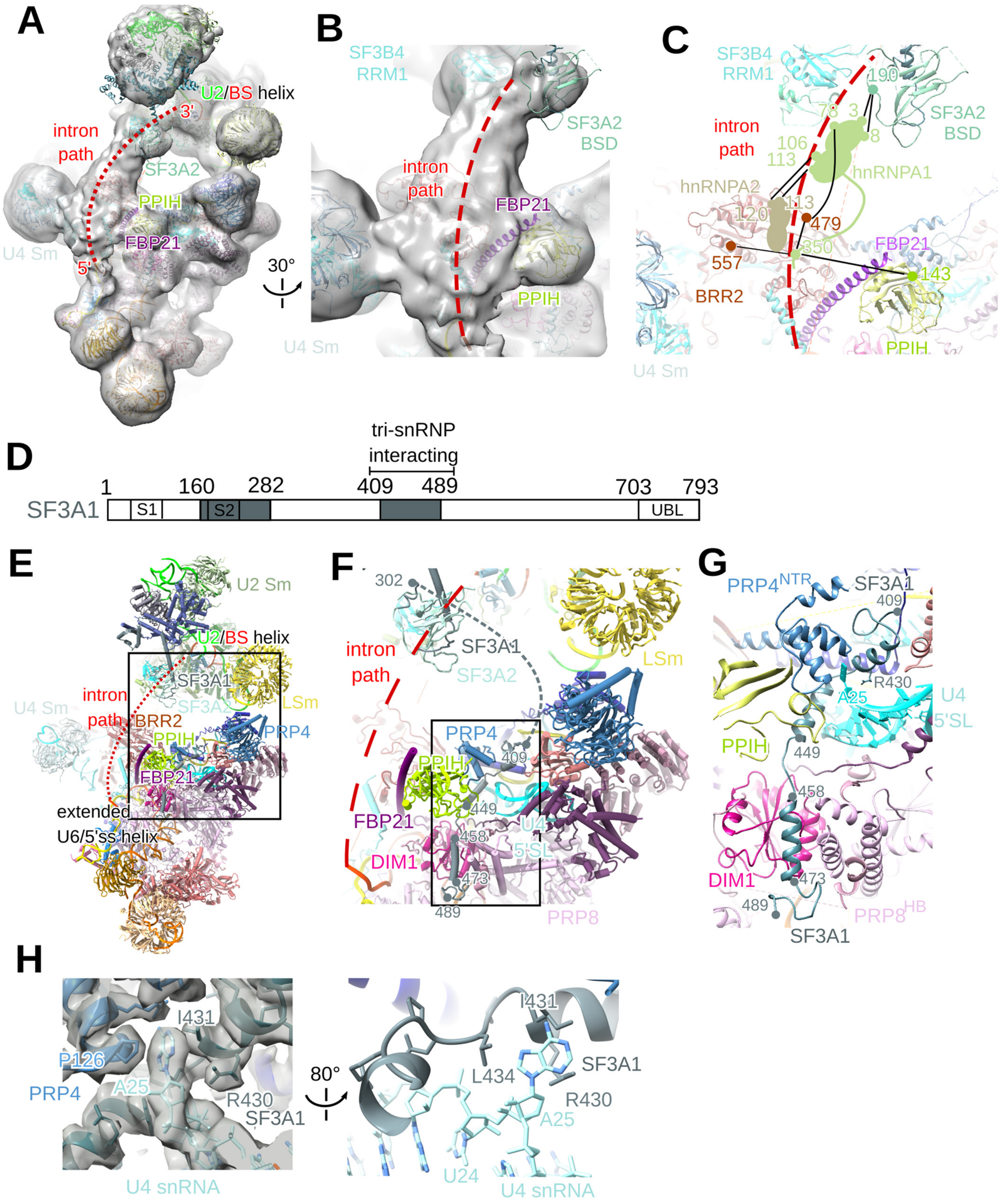
A
U2/BS helix
3'
intron
path
SF3A2
PPIH
FBP21
U4 Sm
5'
30°
B
SF3B4
RRM1
SF3A2
BSD
intron
path
FBP21
PPIH
U4 Sm
C
SF3B4
RRM1
190
SF3A2
BSD
intron
path
hnRNPA1
hnRNPA2
479
FBP21
557
143
BRR2
PPIH
U4 Sm
D
tri-snRNP
interacting
1
160
282
409
489
703
793
SF3A1
S1
S2
UBL
E
U2 Sm
U2/BS helix
SF3A1
SF3A2
LSm
U4 Sm
intron
path
BRR2
PPIH
PRP4
FBP21
extended
U6/5'ss helix
F
302
intron
path
SF3A1
SF3A2
LSm
PRP4
409
PPIH
449
FBP21
458
U4
5'SL
DIM1
473
489
PRP8
G
PRP4NTR
SF3A1
409
R430
A25
U4
5'SL
PPIH
449
458
DIM1
473
489
PRP8HB
SF3A1
H
I431
P126
PRP4
A25
R430
SF3A1
U4 snRNA
80°
I431
SF3A1
L434
R430
A25
U24
U4 snRNA

Figure EV5. Location of intron nucleotides upstream of the branch site and a C-terminal region of SF3A1 in hB.

(**A**) Proposed path (dashed line) of the intron between the U2/BS helix and U6/5′ss helix. (**B**, **C**) Intron nucleotides directly upstream of the BS are sandwiched between SF3B4 RRM1 and the β-sandwich domain (BSD) of SF3A2, and nucleotides further upstream are likely contacted by hnRNP proteins. Panel (**B**) shows the EM density that appears to accommodate the intron, as well as adjacent protein domains. Panel (**C**) shows the proposed position of hnRNPA1 and A2, based on protein crosslinks with neighboring B complex proteins. Intermolecular crosslinks are indicated by a line, with the position of the crosslinked residues indicated. (**D**) Domain organization of SF3A1. The modeled regions are indicated as colored boxes. S1 and S2, SURP domains 1 and 2; UBL, ubiquitin-like domain. (**E**) Overview of the hB complex molecular architecture. The boxed region is expanded in panel (**F**). (**F, G**). SF3A1 aa 409–449 interact with U4 SL1, PRP4 and PPIH, while SF3A1 aa 454–489, which form an α-helix, are located between DIM1 and the PRP8 helical bundle ($PRP8^{HB}$). The region boxed in panel (**F**) is expanded in panel (**G**). (**H**) Fit of indicated U4 nucleotides, and amino acids of PRP4 and SF3A1 into the hB EM density. The base of A25 interacts with several hydrophobic protein side chains (i.e., I431, L434 of SF3A1 and P126 of PRP4), while R430 of SF3A1 contacts the backbone of U4-A25.

© The Author(s)
